# The Effect of Human Milk on Modulating the Quality of Growth in Preterm Infants

**DOI:** 10.3389/fped.2018.00291

**Published:** 2018-10-09

**Authors:** Pasqua Piemontese, Nadia Liotto, Domenica Mallardi, Paola Roggero, Valeria Puricelli, Maria Lorella Giannì, Daniela Morniroli, Chiara Tabasso, Michela Perrone, Camilla Menis, Anna Orsi, Orsola Amato, Fabio Mosca

**Affiliations:** ^1^Neonatal Intensive Care Unit, Fondazione IRCCS Ca' Granda Ospedale Maggiore Policlinico (IRCCS), Milan, Italy; ^2^Department of Clinical Sciences and Community Health, University of Milan, Milan, Italy

**Keywords:** human milk, donor human milk, preterm infants, body composition, target fortification

## Abstract

**Introduction:** Human milk is the optimal nutrition for preterm infants. When the mother's own milk is unavailable, donor human milk is recommended as an alternative for preterm infants. The association among early nutrition, body composition and the future risk of disease has recently attracted much interest. The aim of this study was to investigate the effect of human milk on the body composition of preterm infants.

**Materials and Methods:** Very low birth weight infants (VLBW: birth weight <1,500 g) with a gestational age (GA) between 26 and 34 weeks were included. Clinical data, anthropometric measurements and nutritional intake in terms of the volume of human milk were extracted from computerized medical charts. The human milk intake was expressed as a percentage of target fortified donor human milk and/or target fortified fresh mother's milk, compared with the total volume of milk intake during the hospital stay. All included infants underwent anthropometric measurements and body composition analysis (expressed as fat-free mass percentage) at term corrected age (CA) by air-displacement plethysmography. A comparison between infants fed human milk at <50% (group 1) and infants fed human milk at ≥50% of the total volume of milk intake (group 2) was conducted. Multiple linear regression analyses were conducted to explore the modulating effect of fortified human milk on fat-free mass at term CA.

**Results:** Seventy-three VLBW infants were included in the study. The mean weight and GA at birth were 1,248 ± 198 g and 30.2 ± 2.0 weeks, respectively. No differences were found regarding anthropometric measurements at birth, at discharge and at term CA between the two groups. The mean fortified human milk intake was 34.9 ± 12.5 and 80.9 ± 15.5% in groups 1 and 2, respectively (*p* < 0.001).

A multiple regression analysis corrected for sex and birth weight demonstrated that intake of ≥50% fortified human milk was associated with a higher fat-free mass percentage at term CA than intake of <50% fortified human milk.

**Conclusion:** The use of target fortified human milk modulated growth and improved growth quality in vulnerable preterm infants. Thus, the use of donor human milk should be encouraged when fresh mother's milk is insufficient or not available.

## Introduction

Human milk is the optimal nutrition for preterm infants. Increasing evidence has demonstrated that mother's own milk provides significant benefits for extremely preterm infants, such as the prevention of infection and necrotizing enterocolitis and a reduction in the duration of hospital stay (1). In addition, it has been reported that these benefits are modulated according to the dose of human milk fed during hospitalization (2, 3).

Mother's own milk is at the top of the biological hierarchy; therefore, it is always preferable to other sources of milk (4). Nevertheless, donor human milk can be a bridge until a mother's own milk is available and sufficient to meet her infant's nutritional needs (1, 5). Indeed, in a recent meta-analysis, mother's own milk supplemented with donor human milk was associated with a reduced risk in the development of bronchopulmonary dysplasia in very preterm infants compared with that supplemented with preterm formula (6). In 2014, Quigley and McGuire demonstrated that formula-fed preterm and low birth weight infants had higher rates of short-term growth but also a higher risk of developing necrotizing enterocolitis than those who were fed donor human milk (7).

The association among early nutrition, growth, growth quality, and the future risk of developing noncommunicable diseases has attracted much interest in recent years.

In a recent study on the growth of late preterm infants, we demonstrated that being fed human milk is associated with increased fat-free mass deposition at term corrected age (CA) (8).

It has also been reported that formula-fed very preterm infants show an altered body composition. Specifically, compared with breastfed very preterm infants, formula-fed very preterm infants showed increased fat mass at term CA (9, 10), which decreased toward 6 months CA (11).

Conversely, preterm infants fed fortified mother's own milk showed a higher percentage of fat-free mass than formula-fed infants at term CA (12).

Data regarding the effect of human milk (both fresh mother's milk and/or donor human milk) on the modulation of body composition in preterm infants are scarce. Therefore, the aim of this study was to investigate the effect of human milk (both fresh mother's milk and/or donor human milk) on growth and body composition in preterm infants.

## Materials and methods

The Ethics Committee of the Fondazione Istituto di Ricovero e Cura a Carattere Scientifico Cà Granda Ospedale Maggiore Policlinico approved the study, and written informed consent was obtained from the parents. All clinical investigations were conducted according to the principles outlined in the Declaration of Helsinki.

### Study design

A longitudinal observational study was conducted.

Very low birth weight infants (VLBW: birth weight <1,500 g) born between May 2016 and December 2017 at the authors' institution with gestational age (GA) at birth from 26^+0^ to 33^+6^ weeks were included in the study.

The exclusion criteria for all infants screened included the presence of congenital diseases, chromosomal abnormalities or cardiac, brain, renal, endocrine or surgical diseases, which can interfere with growth. In addition, infants who required ventilatory assistance and intravenous nutritional support at term CA were excluded.

GA was based on the last menstrual period and first trimester ultrasonogram. CA was calculated using the chronological age and adjusted for GA, which is the number of additional weeks from term (40 weeks) (13).

### Data collection and nutritional practices

Clinical data, anthropometric measurements, and nutritional intake in terms of the volume of human milk intake (both fresh mother's milk and/or donor human milk) during the hospital stay were acquired from computerized medical charts (Neocare®).

Parenteral nutrition was started on the first day of life. The parenteral solutions were prepared by the hospital pharmacy according to the medical prescription. The provided volume increased from 80 to 90 ml/kg on the first day to 150–180 ml/kg on the 7th day of life, with an energy/protein ratio from 20.8–24 kcal/g on the first day up to 23.1–27.7 kcal/g on the 7th day of life.

Weaning from parenteral nutrition was scheduled to obtain a weight velocity >15 g/kg/day. To achieve this goal, the macronutrients were reduced gradually according to weight velocity. Table 1 shows the parenteral nutrition practices according to the internal procedure.

**Table 1:** Parenteral nutrition practices.

**Day of life**	**1**		**2**		**3**		**4**		**5**		**6**		≥**7**	
**Birth weight (g)**	**<1000 g**	**>1000 g**	**<1000 g**	**>1000 g**	**<1000 g**	**>1000 g**	**<1000 g**	**>1000 g**	**<1000 g**	**>1000 g**	**<1000 g**	**>1000 g**	**<1000 g**	**>1000 g**
Volume (ml/kg)	80–90	80	90–100	90	110–120	100–110	120–140	110–120	140–160	120–130	150–160	140–150	150–160	150–160
Glucose (g/kg)	8	9	8	9	9	10–11	9	10–13	10	10–14	10–13	10–14	10–14	10–14
Lipids (g/kg)	2–2.5	2.5	2.5	2.5	2.5	2.5	3	3	3	3	3	3	3	3
Proteins (g/kg)	2.5–3	3	3.5	3	3–3.5	3.5	3–3.5	3,5	3.5–4	3,5	4	3,5	4	3,5
Sodium (mEq/kg)	0	0	0	0	0	0	3	3	3	3	3,5	3.5	4	3.5–4
Potassium (mEq/kg)	0	0	0	0	0	0	2	2	2	2	2	2	3	2–4
Cloride (mEq/kg)	0	0	0	0	0	0	3	3	3	3	3.5	3.5	3.5-6	3.5–4
Calcium (mg/kg)	0	0	60	60	60	70	70	80	80	80	80	80	80	90
Phosphate (mg/kg)	0	0	30	30	30	40	40	50	50	55	55	60	60	70
Magnesium (mEq/kg)	0	0	0.5	0.5	0.5	1	0.5	1	0,5	1	0,5	1	0,5	1

*Parenteral nutrition recommendation according to internal procedure (fluids, macronutrients and micronutrients)*.

Enteral feeding was started within 24 h of postnatal life using fresh mother's milk. When fresh mother's milk was unavailable or insufficient, donor human milk was started following the acquisition of written informed consent from the parents. In case of lack of parents' consent, infants were fed with preterm formula (energy: 83 kcal/100 ml; carbohydrates: 8.4 g/100 ml; proteins: 2.7 g/100 ml; fat: 4.1 g/100 ml).

For infants with birth weights less than 1,000 g and/or with severe intrauterine growth restriction (<3th percentile according to INTERGROWTH-21st reference curves) (14), enteral feeding was started at 10 ml/kg/day and remained stable for the first 3 days of life. On the fourth day of life, an increase of 10 ml/kg/day was scheduled.

For infants with birth weights greater than or equal to 1,000 g without severe intrauterine growth restriction (<3th percentile according to INTERGROWTH-21st reference curves) (14), enteral feeding was started at 20 ml/kg/day and remained stable for the first 2 days of life. After the third day of life, an increase of 20 ml/kg/day was scheduled.

Enteral nutrition was stopped in cases of septic shock, needs for exsanguinotrasfusion, abdominal distension with a visible intestinal loop, and gastric residual volumes >2 ml/kg for infants with birth weights <750 g, >3 ml/kg for infants with birth weights ranging from 751 to 1,000 g and >5 ml/kg for infants with birth weights greater than 1,000 g.

When the infants tolerated an enteral intake ≥80 ml/kg, a target human milk fortification was started.

Specifically, both donor pasteurized human milk and fresh mother's milk were analyzed using a mid-ray spectrometry human milk analyzer (Miris AB®).

Pooled donor human milk was created by mixing mature thawed human milk from 1 to 5 donors enrolled at the Human Milk Bank at the authors' institution. Analyses of fresh mother's milk were conducted two times/week using a 10-ml sample from a 24 h fresh milk pool. Analyses of donor human milk were conducted using a 10-ml sample from each pool after Holder pasteurization (62.5°C for 30 min) to account for macronutrient depletion due to storage and pasteurization (15). According to these results, the medical team prescribed individualized fortification to comply with the ESPGHAN nutrient intake guidelines (16).

Human milk fortification was performed using bovine human milk fortifiers: FM85 (Nestlè) or Aptamil BMF (Nutricia) as polymeric fortifiers, Aptamil PS (Nutricia) for protein supplementation, and medium-chain triglyceride (MCT) oil (Medifood) for energy provision.

Human milk intake was expressed as the volume of fortified donor human milk and/or fortified fresh mother's milk.

The percentage of human milk intake was computed from the volume of human milk intake compared with the total volume of milk intake during the hospital stay (fortified donor human milk+fortified fresh mother's milk+formula milk). In addition, the proportion of mothers' own milk within the total volume of human milk intake was computed (fresh mother's milk intake %).

### Anthropometric measurements

Body weight, body length and head circumference of all infants included in the study were assessed at birth and at term CA according to standard procedures (17, 18). In detail, a subject's body weight was measured on an electronic scale accurate to the nearest 0.1 g (PEA POD Infant Body Composition System; COSMED, Italy), body length was measured to the nearest mm using a recumbent infant length board, and head circumference was measured to the nearest 1 mm using a nonstretch measuring tape.

Infants with birth weights below the 10th percentile according to INTERGROWTH-21st reference curves (14) were categorized as small for gestational age (SGA).

The weight z-scores at birth and at discharge were calculated according to INTERGROWTH-21st reference and standard curves, respectively (14, 18).

The daily growth rate was calculated using the exponential regression model described by Patel et al. (19) as follows:

(1) $$\begin{array}{l} \left[1000\times ln\left(W2/W1\right)\right]/\left(D2-D1\right) \end{array}$$

where W = weight in grams; D = day; 1 = beginning of the time interval; and 2 = end of the time interval (19).

### Body composition assessment

Body composition was assessed at term CA for all infants included in the study using an air-displacement plethysmography system (PEA POD Infant Body Composition System; COSMED, Italy) (20, 21). The PEA POD assesses FM and FFM by body mass and body volume measurements via the application of whole-body densitometric principles. Body density was computed from the study subject's measured mass and volume and then converted to indicate the total absolute (g) and percentage (%) of FM and FFM using sex-specific equations developed by Fomon et al. (22). The observers were health professionals trained to perform anthropometric and body composition measurements according to our standard procedure. The interobserver coefficient of variation for the FM percentage estimates was 0.3%.

### Statistical analysis

Continuous variables are reported as the mean and standard deviation (SD). Categorical variables are reported as absolute numbers or percentages. Comparisons between infants fed human milk (fresh mother's milk or donor human milk) at less than 50% of the total volume of milk intake during the hospital stay (group 1) and infants fed human milk at greater or equal to 50% of the total volume of milk intake during the hospital stay (group 2) were conducted using the X^2^ test for discrete variables and analysis of variance for continuous variables.

Multiple linear regression analyses were conducted to explore the role of fortified human milk in modulating fat-free mass at term CA according to sex and birth weight. Indeed, it has been reported that gender can influence body composition at term CA (23).

Statistical significance was set at a level of 0.05. All statistical analyses were performed using SPSS software (SPSS, version 20; SPSS, Chicago, IL).

## Results

Seventy-three VLBW infants were included in the study. The basic characteristics at birth are shown in Table 2. No differences were detectable at birth between the two study groups.

**Table 2:** Basic characteristics at birth of infants included.

	**All infants (*n* = 73)**	**Group 1 (*n* = 24)**	**Group 2 (*n* = 49)**	***p***
Weight (g)	1248 ± 198	1207 ± 208	1269 ± 193	0.215
Length (cm)	38.3 ± 2.8	37.7 ± 3.3	38.6 ± 2.6	0.278
Head Circumference (cm)	26.8 ± 1.9	26.5 ± 2.3	27.0 ± 1.7	0.358
Gestational age (weeks)	30.2 ± 2.0	30.0 ± 2.4	30.3 ± 1.8	0.632
Males: n (%)	32 (43.9)	10 (41.6)	22 (44.9)	0.797
Twins: n (%)	35 (48.5)	13 (54.1)	22 (44.9)	0.463
SGA infants n (%)	25 (34.2)	9 (37.5)	14 (28.6)	0.305
Antenatal steroids n (%)	70 (95.9)	23 (95.8)	47 (95.9)	0.704
Need of invasive ventilatory assistance n (%)	17 (23.3)	5 (20.8)	12 (24.5)	0.558
Duration of invasive ventilatory assistance (days)	0.88 ± 2.0	0.61 ± 1.7	1.0 ± 2.1	0.446
Need of non-invasive ventilatory assistance n (%)	41 (56.1)	15 (62.5)	26 (53.1)	0.558
Duration of invasive ventilatory assistance (days)	20.94 ± 21.0	19.74 ± 23.7	21.53 ± 19.8	0.741

*Data are expressed as mean ± standard deviation or n (%). The duration of ventilatory assistance were expressed as days of treatment*.

The infants included in the study received parenteral nutrition for 21.9 ± 9.0 days, and full enteral feeding was achieved at 28.9 ± 9.0 days of life; no differences were found between the two groups.

Data regarding human milk intake (fresh mother's milk and/or pasteurized donor human milk) are shown in Table 3.

**Table 3:** Milk volume intake during hospital stay.

	**All infants (*n* = 73)**	**Group 1 (*n* = 24)**	**Group 2 (*n* = 49)**	***p***
Total Milk volume intake (ml)	10357.1 ± 5871.7	11713.1 ± 7756.7	9692.9 ± 4638.5	0.169
Human milk daily intake (ml/day)	118.2 ± 47.3	65.6 ± 25.9	144.0 ± 31.2	<0.001
Human milk volume intake (ml)	6385.4 ± 3498.9	3998.6 ± 2590.8	7554.4 ± 3302.1	<0.001
Human milk volume intake (%)	65.8 ± 26.1	34.9 ± 12.5	80.9 ± 15.5	<0.001
Fresh mother milk intake (%)	64.7 ± 42.6	41.9 ± 43.7	75.9 ± 37.6	0.001

*Data are expressed as mean ± standard deviation. The percentage of human milk intake was computed from the volume of human milk intake compared with the total volume of milk intake during the hospital stay (fortified donor human milk + fortified fresh mother's milk+formula milk). Fresh mother milk intake (%) is the proportion of mothers' own milk within the total volume of human milk*.

The mean energy and protein intake were 65.7 ± 16.4 Kcal/kg/day and 2.9 g/kg/day during the first week of life, 124.8 ± 13.8 kcal/kg/day and 3.3 ± 0.7 g/kg/day when full enteral feeding was achieved, and 130.8 ± 14.2 kcal/kg/day and 3.6 ± 0.6 g/kg/day at discharge, respectively. No differences in energy and protein intake between the two study groups were detectable during the hospital stay.

The weight z-score at birth and at discharge and the daily growth rates are shown in Table 4.

**Table 4:** Weight z-score and daily growth rate during hospital stay.

	**All infants (*n* = 73)**	**Group 1 (*n* = 24)**	**Group 2 (*n* = 49)**	***p***
Birth weight z-score	−0.60 ± 0.97	−0.61 ± 1.2	−0.59 ± 0.84	0.941
Weight at discharge z-score	−1.26 ± 1.01	−1.14 ± 1.27	−1.32 ± 0.87	0.504
Daily growth rate during parenteral nutrition (g/kg/day)	20.23 ± 5.38	21.99 ± 4.35	19.4 ± 5.67	0.051
Daily growth rate during full enteral feeding (g/kg/day)	15.32 ± 8.06	18.65 ± 12.24	13.70 ± 4.17	0.013
Total daily growth rate during hospital stay (g/kg/day)	16.07 ± 2.42	17.30 ± 2.74	15.46 ± 2.01	0.002

*Data are expressed as mean ± standard deviation. The daily growth rates were calculated during the administration of parenteral nutrition, after reaching full enteral feeding (milk volume intake ≥150 ml/kg/day) and during all the hospital stay*.

No differences were found in weight z-score at birth or at discharge between the two groups. Group 1 had a higher growth rate than group 2 both after reaching full enteral feeding (milk volume intake≥150 ml/kg/day) and during the entire hospital stay.

The incidence of comorbidities developed during hospitalization was similar in group 1 and group 2. Among all infants included in the study, none developed NEC, 9.6% had cholestasis, 5.5% had bronchopulmonary dysplasia, 9.6% received treatment for patent ductus arteriosus, and 17.8% had sepsis.

The characteristics at discharge and at term CA according to the mode of feeding are shown in Tables 5, 6. No differences were found between the two groups regarding the anthropometric measurements and body composition at discharge or at term CA. The infants included in the study achieved the ability to feed orally beginning at 36.4 ± 1.4 weeks of postmenstrual age. No differences were found between groups.

**Table 5:** Characteristics at discharge according to mode of feeding.

	**All infants (*n* = 73)**	**Group 1 (*n* = 24)**	**Group 2 (*n* = 49)**	***p***
Weight (g)	2404 ± 424	2471 ± 514	2371 ± 373	0.349
Length (cm)	45.1 ± 2.2	45.3 ± 2.2	44.9 ± 2.2	0.544
Head Circumference (cm)	32.0 ± 2.0	32.2 ± 1.7	31.9 ± 2.1	0.604
Postmenstrual age (weeks)	37.7 ± 1.5	37.8 ± 1.4	37.6 ± 1.6	0.470
Length of stay (days)	51.9 ± 18.5	52.8 ± 19.4	51.4 ± 18.1	0.763

*Data are expressed as mean ± standard deviation*.

**Table 6:** Anthropometric measurements and body composition at term corrected age according to mode of feeding.

	**All infants (*n* = 73)**	**Group 1 (*n* = 24)**	**Group 2 (*n* = 49)**	***p***
Weight (g)	3088 ± 476	3142 ± 628	3062 ± 386	0.506
Length (cm)	48.5 ± 2.3	48.4 ± 2.7	48.6 ± 2.1	0.774
Head Circumference (cm)	34.6 ± 1.3	34.4 ± 1.7	34.7 ± 1.1	0.346
Fat free mass (%)	82.0 ± 11.1	78.6 ± 18.1	83.7 ± 4.5	0.067

*Data were expressed ad mean ± standard deviation*.

Multiple logistic regression analysis showed that there was a positive association between the human milk volume intake percentage and fat-free mass percentage after correction for birth weight and gender (β = 0.12 ± 0.05, *p* = 0.01). Specifically, the regression model showed that being fed with human milk at more than or equal to 50% of total milk intake was associated with a significant increase in fat-free mass percentage at term CA (Table 7).

**Table 7:** Multiple regression analysis model for fat free mass %.

	**Beta coefficients**	**95% Interval of confidence**	***p***
Intercept	95.518 ± 7.876	79.805; 111.231	<0.001
Being male (no vs. yes)	4.396 ± 2.481	−0.552; 9.345	0.081
Birth weight (g)	−0.01 ± 0.006	−0.028; −0.003	0.016
Being fed human milk ≥50% (no vs. yes)	5.883 ± 2.638	0.621; 11.146	0.029

*Multiple regression analysis model corrected by gender and birth weight. Being fed with human milk more than or equal to 50% was positively associated with a significant increase in fat free mass percentage at term CA*.

## Discussion

This study examined the relationship between the type of feeding and body composition in VLBW infants. Specifically, we demonstrated that VLBW infants fed human milk (both fresh mother's milk and/or donor human milk) at more than or equal to 50% of the total milk volume intake during the hospital stay displayed an increased fat-free mass percentage at term CA compared to VLBW infants fed human milk at less than 50% of the total milk intake.

Johnson et al. demonstrated that VLBW infants had a fat-free mass deficit at term CA compared to full-term infants (24). Increasing evidence has demonstrated that greater fat-free mass deposition is associated with an improved neurodevelopmental outcome (25). A study conducted at our center demonstrated that human milk feeding is positively associated with fat-free mass deposition in late preterm infants (8).

Our results showed that infants fed human milk at less than 50% of the total milk intake had a higher growth rate than their counterparts. On the other hand, the weight and weight z-score at discharge were similar between the two groups. It must be noted that most of the infants fed fresh mother's milk were in group 2, and consequently, the higher number of breastfed infants can explain the lower growth rate in these infants than in formula-fed infants. Indeed, after reaching full enteral feeding and, above all, after reaching the ability to feed orally, breastfed infants cannot receive adequate fortification from feedings taken directly from the breast. A similar weight was found at term CA between the two groups. Indeed, considering the weight increase percentage between discharge and term CA, infants in group 2 show a slightly higher value than those in group 1 (+31.4 vs. 28.3%), probably due to better adaptation of group 2 infants than group 1 infants after discharge.

To the best of our knowledge, this was the first study to explore the modulating effect of both fresh mother's milk and donor human milk on body composition. In a previous study conducted in our center, Morlacchi et al. demonstrated that preterm infants fed fortified mother's own milk showed a higher percentage of fat-free mass than formula-fed infants at term CA, which was probably due to the higher nitrogen balance in breastfed infants than in formula-fed infants (12). In our study, we did not evaluate infant nitrogen balance; however, we can speculate that being fed both fortified fresh mother's milk and/or donor human milk can result in a similar metabolic response to that obtained by feeding exclusively fresh mother's milk. In addition, in the univariate regression analysis, the percentage of fresh mother's milk intake was not associated with FFM deposition, whereas the total human milk volume intake was positively associated with the FFM percentage at term CA. This observation can most likely be explained by the relatively low percentage of fresh human milk ingested by infants included in the study.

It has been reported that exclusive breastfeeding is associated with lower growth rates in preterm infants than formula feeding (26), especially with standard fortification of human milk (27). In fact, the mean protein content decreases during lactation, while the nutrient needs of VLBW infants remain high (28, 29). In addition, although it has been reported that donor human milk provides nutrients comparable to mother's milk, preterm infants fed donor human milk fortified similarly to fresh mother's milk according to standard methods showed an increased risk of postnatal growth restriction (30, 31). In contrast, it has been reported that fortification of donor human milk to reach 3.5 g/kg of protein intake is associated with significantly greater weight gain and head growth in VLBW infants than feeding a formula-based diet (30). In addition, targeted fortification of human milk seems to represent an optimal strategy to prevent postnatal growth restriction (32, 33).

According to our internal procedure, when infants tolerated an enteral intake of human milk (both fresh mother's milk and/or donor human milk) ≥80 ml/kg/day, targeted human milk fortification is initiated to meet the ESPGHAN guidelines. Consequently, infants fed fortified human milk at more than or equal to 50% of the total milk volume intake during the hospital stay exhibited similar growth at discharge as infants fed less human milk.

In a recent study of 34 VLBW infants (11 breastfed and 23 formula-fed) compared with a control group of 19 full-term infants, it was demonstrated that formula-fed infants were heavier than breastfed infants at term CA and showed higher amounts of adipose tissue and lower amounts of fat-free mass than full-term infants. However, breastfed preterm infants had a similar body composition as full-term infants (34, 35).

In our study, we did not observe any difference in body weight between the two groups, irrespective of the type of feeding, both at discharge and at term CA. Nevertheless, infants in group 2 who were fed ~81% human milk during their hospital stay had an increased fat-free mass at term CA compared with infants in group 1 who were fed only 35% human milk from birth to discharge.

It has been taken into account that in our study, the donor human milk constituted 58% and 25% of the total amount of human milk intake in group 1 and group 2, respectively. This detail underlines the important effect of fresh mother's milk on growth and quality of growth but suggests that donor human milk is preferred over formula milk when fresh mother's milk is not available or is insufficient to satisfy the infant's nutritional needs. Therefore, the strength of the study was the use of donor human milk to supplement fresh mother's milk and the practice of targeted fortification to achieve the nutritional needs of preterm infants.

It must also be taken into account that the infants included in this study were all clinically stable at term CA during the assessment of body composition, and therefore, the results obtained in this study cannot be applied to sick infants. In addition, a limitation of the study is that it is not a randomized controlled trial, although this type of study design has ethical issues.

In conclusion, the use of target fortified human milk modulated the growth of and improved the quality of growth in vulnerable preterm infants. The use of fortified donor human milk when fresh mother's milk is insufficient or not available should be encouraged.

## Author contributions

PP and NL conceived and designed the study and wrote the article. DoM, CT, and MP collected the data and were responsible for database management. MG, CM, AO, DaM, and OA performed the clinical evaluations and contributed to the discussion of the results and PR, VP, and FM provided suggestions concerning the content and concept of the article and were responsible for critically revising the manuscript.

### Conflict of interest statement

The authors declare that the research was conducted in the absence of any commercial or financial relationships that could be construed as a potential conflict of interest.

## References

[1] Maffei D Schanler RJ. Human milk is the feeding strategy to prevent necrotizing enterocolitis! Semin Perinatol. (2017) 41:36–40. 10.1053/j.semperi.2016.09.016 27836421

[2] Cacho NT Parker LA Neu J. Necrotizing enterocolitis and human milk feeding: a systematic review. Clin Perinatol. (2017) 44:49–67. 10.1016/j.clp.2016.11.009 28159209

[3] Miller J Tonkin E Damarell RA McPhee AJ Suganuma M Suganuma H. A systematic review and meta-analysis of human milk feeding and morbidity in very low birth weight infants. Nutrients (2018) 10:6 10.3390/nu10060707 29857555 PMC6024377

[4] Committee on nutrition; Section on breastfeeding; Committee on fetus and newborn Donor Human Milk for the High-Risk Infant: Preparation, Safety, and Usage Options in the United States. Pediatrics (2017) 139:1 10.1542/peds.2016-3440 27994111

[5] Parker MG Burnham L Mao W Philipp BL Merewood A. Implementation of a donor milk program is associated with greater consumption of Mothers' own milk among VLBW Infants in a US, Level 3 NICU. J Hum Lact. (2016) 32:221–8. 10.1177/0890334415598305 26243756

[6] Villamor-Martínez E Pierro M Cavallaro G Mosca F Kramer BW Villamor E. Donor human milk protects against bronchopulmonary dysplasia: a systematic review and meta-analysis. Nutrients (2018) 10:2. 10.3390/nu10020238 29461479 PMC5852814

[7] Quigley M McGuire W Formula versus donor breast milk for feeding preterm or low birth weight infants. Cochrane Database Syst Rev. (2014) 2014:CD002971. 10.1002/14651858.CD002971.pub3 24752468

[8] Giannì ML Consonni D Liotto N Roggero P Morlacchi L Piemontese P. Does human milk modulate body composition in late preterm infants at term-corrected age? Nutrients (2016) 8:E664. 10.3390/nu8100664 27782098 PMC5084050

[9] Huang P Zhou J Yin Y Jing W Luo B Wang J. Effects of breast-feeding compared with formula-feeding on preterm infant body composition: a systematic review and meta-analysis. Br J Nutr. (2016) 116:132–41. 10.1017/S0007114516001720 27181767

[10] Cooke RJ Griffin IJ McCormick K Adiposity is not altered in preterm infants fed with a nutrient-enriched formula after hospital discharge. Pediatr Res. (2010) 67:660–4. 10.1203/PDR.0b013e3181da8d01 20216105

[11] Gale C Logan KM Santhakumaran S Parkinson JRC Hyde MJ Modi N. Effect of breastfeeding compared with formula feeding on infant body composition: a systematic review and meta-analysis. Am J Clin Nutr. (2012) 95:656–69. 10.3945/ajcn.111.027284 22301930

[12] Morlacchi L Roggero P Giannì ML Bracco B Porri D Battiato E Protein use and weight-gain quality in very-low-birth-weight preterm infants fed human milk or formula. Am J Clin Nutr. (2018) 107:195–200. 10.1093/ajcn/nqx001 29529139

[13] Engle WA American academy of paediatrics committee on foetus and newborn. Age terminology during the perinatal period. Paediatrics (2004) 114:1362–4. 10.1542/peds.2004-1915 15520122

[14] Villar J Giuliani F Fenton TR Ohuma EO Ismail LC Kennedy SH Intergrowth-21st Consortium. Intergrowth-21st very preterm size at birth reference charts. Lancet (2016) 387:844–45. 10.1016/S0140-6736(16)00384-6 26898853

[15] Vieira AA Soares FV Pimenta HP Abranches AD Moreira M.E. Analysis of the influence of pasteurization, freezing/thawing, and offer processes on human milk's macronutrient concentrations. Early Hum Dev. (2011) 87:577–80. 10.1016/j.earlhumdev.2011.04.016 21592688

[16] Agostoni C Buonocore G Carnielli VP De Curtis M Darmaun D Decsi T. Enteral nutrient supply for preterm infants: commentary from the European Society of Paediatric Gastroenterology, Hepatology and Nutrition Committee on Nutrition. J Pediatr Gastroenterol Nutr. (2010) 50:85–91. 10.1097/MPG.0b013e3181adaee0 19881390

[17] Agostoni C Grandi F Giannì ML Silano M Torcoletti M Giovannini M. Growth patterns of breast fed and formula fed infants in the first 12 months of life: an Italian study. Arch Dis Child (1999) 81:395–9. 10519710 10.1136/adc.81.5.395 PMC1718130

[18] Villar J Cheikh IL Victora CG Ohuma EO Bertino E Altman DG. International standards for newborn weight, length, and head circumference by gestational age and sex: the Newborn Cross-Sectional Study of the INTERGROWTH-21st Project. Lancet (2014) 384:857–68. 10.1016/S0140-6736(14)60932-6 25209487

[19] Patel AL Engstrom JL Meier PP Jegier BJ Kimura RE. Calculating postnatal growth velocity in very low birth weight (VLBW) premature infants. J Perinatol. (2009) 29:618–22. 10.1038/jp.2009.55 19461590 PMC2767524

[20] Ma G Yao M Liu Y Lin A Zou H Urlando A Validation of a new paediatric air displacement plethysmograph for assessing body composition in infants. Am J Clin Nutr. (2004) 79:653–60. 10.1093/ajcn/79.4.653 15051611

[21] Ellis KJ Yao M Shypailo RJ Urlando A Wong WW Heird WC. Body composition assessment in infancy: air-displacement plethysmography compared with a reference 4-compartment model. Am J Clin Nutr. (2007) 85:90–5. 10.1093/ajcn/85.1.90 17209182

[22] Fomon SJ Haschke F Ziegler EE Nelson SE. Body composition of reference children from birth to age 10 years. Am J Clin Nutr. (1982) 35:1169–75. 10.1093/ajcn/35.5.1169 7081099

[23] Liotto N Roggero P Bracco B Menis C Morniroli D Perrone M Giannì ML Mosca F. Can basic characteristics estimate body composition in early infancy? J Pediatr Gastroenterol Nutr. (2018) 66:e76–e80. 10.1097/MPG.0000000000001758 28953532

[24] Johnson MJ Wootton SA Leaf AA Jackson AA. Preterm birth and body composition at term equivalent age: a systematic review and meta-analysis. Pediatrics (2012) 130:e640–9. 10.1542/peds.2011-3379 22891222

[25] Ramel SE Gray HL Christiansen E Boys C Georgieff MK Demerath EW Greater early gains in fat-free mass, but not fat mass, are associated with improved neurodevelopment at 1 year corrected age for prematurity in very low birth weight preterm infants. J Pediatr. (2016) 173:108–15. 10.1016/j.jpeds.2016.03.003 27056450

[26] Spiegler J Preuß M Gebauer C Bendiks M Herting E Göpel W On behalf of the german neonatal network. does breastmilk influence the development of bronchopulmonary dysplasia? J Pediatr. (2016) 169:76–80. 10.1016/j.jpeds.2015.10.080 26621048

[27] Corvaglia L Aceti A Paoletti V Mariani E Patrono D Ancora G. Standard fortification of preterm human milk fails to meet recommended protein intake: bedside evaluation by near-infrared-reflectance-analysis. Early Hum Dev. (2010) 86:237–40. 10.1016/j.earlhumdev.2010.04.001 20447779

[28] Gidrewicz DA Fenton TR. A systematic review and meta-analysis of the nutrient content of preterm and term breast milk. BMC Pediatr. (2014) 14:216. 10.1186/1471-2431-14-216 25174435 PMC4236651

[29] Ziegler EE. Breast-milk fortification. Acta Paediatr. (2001) 90:720–3. 10.1111/j.1651-2227.2001.tb02795.x 11519972

[30] Newkirk M Shakeel F Parimi P Rothpletz-Puglia P Patusco R Marcus AF. Comparison of calorie and protein intake of very low birth weight infants receiving mother's own milk or donor milk when the nutrient composition of human milk is measured with a breast milk analyzer. Nutr Clin Pract. (2018) 33:679–86. 10.1002/ncp.10060 29603403

[31] Brownell EA Matson AP Smith KC Moore JE Esposito PA Lussier MM. Dose-response relationship between donor human milk, Mother's own milk, preterm formula, and neonatal growth outcomes. J Pediatr Gastroenterol Nutr. (2018) 67:90–6. 10.1097/MPG.0000000000001959 29543698

[32] Ginovart G Gich I Gutiérrez A Verd S. A fortified donor milk policy is associated with improved in-hospital head growth and weight gain in very low-birth-weight infants. Adv Neonatal Care (2017) 17:250–7. 10.1097/ANC.0000000000000387 28749825

[33] Rochow N Fusch G Choi A Chessell L Elliott L McDonald K. Target fortification of breast milk with fat, protein, and carbohydrates for preterm infants. J Pediatr. (2013) 163:1001–7. 10.1016/j.jpeds.2013.04.052 23769498

[34] Morlacchi L Mallardi D Giannì ML Roggero P Amato O Piemontese P. Is targeted fortification of human breast milk an optimal nutrition strategy for preterm infants? An interventional study. J Transl Med. (2016) 14:195. 10.1186/s12967-016-0957-y 27370649 PMC4930619

[35] Nina M Magdalena Z Przemko K. Does type of feeding affect body composition in very low birth weight infants? - A prospective cohort study. Pediatr Neonatol. (2018). [Epub ahead of print]. 10.1016/j.pedneo.2018.04.010 29784603

